# ASSOCIATIONS BETWEEN DSM-5 PERSONALITY PATHOLOGY AND HORNEY’S INTERPERSONAL TRENDS AMONG OLDER ADULTS

**DOI:** 10.1093/geroni/igz038.581

**Published:** 2019-11-08

**Authors:** Daniel L Segal, Lisa E Stone, Frederick L Coolidge, Gabrielle Krus

**Affiliations:** 1 University of Colorado at Colorado Springs, Colorado Springs, Colorado, United States; 2 University of Colorado, Colorado Springs, Colorado, United States

## Abstract

Introduction: The Personality Inventory for DSM-5 (PID-5) is a measure of the alternative model of personality disorders with limited evidence of validity among older adults. This study examined validity of the model through associations with the Horney-Coolidge Tridimensional Inventory (HCTI). Method: Older adults (N=125) completed the PID-5 and the HCTI. Results: Zero-order correlations were computed between the PID-5’s five domains (Negative Affect, Detachment, Antagonism, Disinhibition, and Psychoticism) and the HCTI’s three domains (Compliance, Aggression, and Detachment). Compliance was moderately negatively correlated with Detachment (r = -.27), as expected. Aggression was significantly positively related to all five PID-5 domains and was most strongly correlated with Antagonism (r = .56), Psychoticism (r = .48), and Disinhibition (r = .32). As predicted, Horney’s Detachment was most strongly related to the PID-5’s Detachment (r = .48). Regression analyses were also conducted with PID-5 domains predicting each HCTI type. The Compliance model was significant, with PID-5 domains predicting 13% of variability in Compliance. Negative Affect (positive) and Detachment (negative) were significant predictors. The Aggression model was also significant, with the PID-5 domains accounting for 40% of variability. Antagonism was the only significant positive predictor. Lastly, the Detachment model was significant, with the PID-5 domains predicting 29% of variance in Detachment scores. Negative Affect (negative) and Detachment (positive) were significant predictors. Discussion: Results indicate that the two measures of personality pathology generally converge regarding theoretically similar constructs and diverge around dissimilar domains, providing evidence of validity of the PID-5 for its ability to capture personality traits.

